# Platelet-rich plasma treatment for talar cartilage repair: a systematic review and meta-analysis

**DOI:** 10.1186/s12891-023-06466-y

**Published:** 2023-05-09

**Authors:** Jialei Peng, Qian Wang, Yang Xu, Hongchen He

**Affiliations:** 1 Department of Rehabilitation Medicine, Institute of Rehabilitation Medicine, West China Hospital, Sichuan University, #37 Guoxue Street, Wuhou District, Chengdu, Sichuan 610041 P. R. China; 2 School of Rehabilitation Sciences, West China School of Medicine, Sichuan University, Chengdu, 610041 P. R. China; 3 Rehabilitation Medicine Key Laboratory of Sichuan Province, Chengdu, 610041 P. R. China

**Keywords:** Platelet-rich plasma, Osteochondral lesion of talus, Cartilage repair, Osteoarthritis, Systematic review, Meta-analysis

## Abstract

**Purpose:**

To systematically review the studies regarding to the safety, efficacy and application methods of PRP in promoting the talar cartilage repair.

**Methods:**

A systematic review was performed by searching PubMed, Web of Science, OVID and EMBASE to identify studies that compared the clinical efficacy of PRP for talar cartilage repair. Main outcome was the American Orthopedic Foot and Ankle Society (AOFAS) score for function and Visual Analog Scale (VAS) for pain was the second outcome.

**Results:**

A total of 10 studies were included in this systematic review, including 4 randomized controlled trials, 1 controlled trial, 3 case series and 2 cohort studies. Four RCTs were analyzed using meta-analysis. For all outcomes, statistical results favored PRP group (AOFAS: MD = 7.84; 95% CI= [-0.13, 15.80], I^2^ = 83%, *P* < 0.01; VAS: MD = 1.86; 95% CI= [0.68, 3.04], I^2^ = 85%, *P* < 0.01). There were almost no reports of adverse events related to PRP intervention. Subgroup analysis showed that whether PRP was used alone or combined with other treatments could result in high heterogeneity but no more specific factors were identified to contribute to this.

**Conclusion:**

PRP is safe and effective for talar cartilage repair. In addition to the standardization of PRP preparation and application, it is necessary to distinguish the effects of PRP used alone or in combination with other treatments. In PRP studies, surgical treatment of talar cartilage repair remains the mainstream. The regulation of PRP in surgical applications are worth exploring. The most relative component is the mesenchymal stem cell because it is the only exposed chondrocyte precursor in the articular cavity whether it is microfracture or cell transplantation.

**Trial registration:**

The study was registered in the PROSPERO International prospective register of systematic reviews (CRD42022360183).

## Introduction

The ankle is highly susceptible to physical injuries which may lead to the involvement of the articular surface, ranging from osteochondral lesions of the talus (OLT) to the development of post-traumatic osteoarthritis (OA) [1, 2]. Osteochondral lesion of the talus (OLT) is an area of abnormal, fractured, or damaged cartilage and bone on the articular surfaces of the talus, most commonly on the anterolateral and posteromedial aspects [3]. Osteoarthritis (OA) is characterized by progressive loss of articular cartilage, subchondral bone sclerosis, osteophyte formation and synovial inflammation [4]. Osteoarthritis can progress from talus cartilage lesions [4, 5]. Both two diseases are related to talar cartilage and contribute to clinical symptoms including activity limitation and pain. Ankle OA in particular has been estimated to affect approximately 1% of the population [6]. Three types of cartilage exist in the human body including hyaline cartilage, elastic cartilage and fibrous cartilage [5, 7]. Articular cartilage of ankle is hyaline cartilage which cushions the loading of the joint. Injuries to the articular cartilage can lead to the development of degenerative joint diseases such as osteoarthritis (OA) [5].

Nonoperative treatment of talar cartilage includes activity modification, protected weight-bearing, physical therapy, bracing, and use of nonsteroidal anti-inflammatory drugs [8, 9]. Compared with conservative treatment and surgical treatment, tissue regeneration technology has the characteristics of less trauma and faster repair, attracting more and more attention.

Platelet-rich plasma (PRP) is a bioactive component containing concentrated platelet. PRP contains both pro-inflammatory cytokines and anti-inflammatory cytokines. Pro-inflammatory cytokines such as inter-leukin-1 (IL-1) and tumor necrosis factor α (TNF α) play a key role in cartilage catabolism for they can induce cells in the joint to produce matrix metalloproteinases (MMPs) that in turn are responsible for degradation of the cartilage matrix [7, 10, 11]. Growth factors heal bone and soft tissue through hematoma formation, proliferation and differentiation of mesenchymal cells, chemotaxis, remodeling of inflammatory cells, angiogenesis and formation of extracellular matrix [12, 13]. In the knee, PRP has been used in patients with injuries of articular cartilage, ligament and meniscus, and has been proved effective. Furthermore, leukocyte-poor PRP may be a superior line of treatment for knee OA over leukocyte-rich PRP [14, 15].

Currently, the research and application of PRP in the field of foot and ankle are mainly ankle osteoarthritis and talar cartilage injury, followed by plantar fasciitis, achilles tendinopathy and antero-inferior tibiofibular ligaments. Even though the use of PRP in foot and ankle is increasing, there are no clear indications and no high level of evidence to guide treatment [3, 13]. The existing review of PRP treatment of talar cartilage does not distinguish the superiority of PRP used alone or used in combination with other treatment, and their focuses are different from biomarkers to function. Therefore, the aim of this paper is to summarize the existing research progress of PRP regeneration and repair of talus cartilage and to summarize the research limitations and unsolved problems, then explore the relationship between talus cartilage repair and PRP according to the characteristics of cartilage metabolism.

## Methods

### Search strategy

A systematic search for articles reporting talar cartilage treatment with PRP was conducted using the PubMed, Web of Science, OVID and EMBASE databases from inception to 7 July 2022. The review followed the Preferred Reporting Items for Systematic Reviews and Meta-analysis (PRISMA) guidelines. Two researchers independently (JP, QW) conducted the search progress and screened the titles, abstracts and full texts of the papers. Search terms included a combination of database-specific controlled vocabulary terms or Mesh terms and free-text terms relating to talar cartilage (e.g. ‘osteochondral’ or ‘osteochondral lesion of talus’ or ankle osteoarthritis) and PRP (e.g. ‘platelet rich plasma’). A standardized data collection form to determine whether papers were appropriate for inclusion was used.

### Selection criteria

Cohort, controlled trials, case series, randomized control studies were included. The inclusion and exclusion criteria of the studies were based on the principles of PICO method (population, intervention, comparison, outcome, as followed). Articles published in non-English, in protocol form or with no full text, animal studies and in vitro studies had been excluded. In addition, the literature was also searched manually from the reference list of the articles found in the search of the electronic databases.

#### Population

The target population was characterized with the diagnosis of osteoarthritis of ankle or osteochondral lesions of talus or other problem needed talar cartilage repair.

#### Intervention

The intervention must contain PRP.

#### Comparison

The comparison was placebo or no PRP.

#### Outcome

Function was the main outcome which was measured by the American Orthopedic Foot and Ankle Society (AOFAS) score. The Visual Analogue Scale (VAS) was the second outcome to measure pain intensity.

### Data extraction

Data from the included studies were extracted into a standard form, detailing the author(s), publication year, country, study type, study design, sample size, control or comparison group selection, interventions, and PRP-related data (such as platelet concentration, leukocyte status, and injection method). Besides, intervention method, symptoms duration, BMI, and mean age of each study were extracted for subgroup analysis. Consensus about detailed instructions for screening of abstracts and full texts, risk of bias, quality of assessments of PRP for talar cartilage repair, and data extraction were achieved. Two methodologically trained reviewers applied the consensus to screen study reports for eligibility and extracted data independently.

### Quality assessment

Cochrane Handbook for Systematic Reviews of Interventions [16] was used to assess the quality of selected RCT studies. Different colors (green, red, yellow) and symbols “+”, “-”, “?”) were used to denote “low risk bias”, “high risk bias” and “unclear bias”. For each criterion, studies were judged to be at either high or low risk of bias. Studies with a high risk of bias for 3 or more criteria were classified as being at high risk of bias overall. The Newcastle-Ottawa scale (NOS) was used to assess the quality of selected cohort studies by 3 indicators: selection, comparability and outcome. Studies scoring ≥ 5 and ≤ 8 were designated low risk of bias, ≥ 3 and ≤ 4 as moderate and ≤ 2 as high.

### Data synthesis and analysis

A meta-analysis was conducted via Revman 5.3 for all outcomes in which at least 2 comparisons were available. Forest plot was used to display results. Only RCTs could enter into meta-analysis. All indicators were continuous outcomes, thus were summarized as means and SDs. Defects were expressed as mean differences and 95% CIs. Data were interpreted in light of changes in variables. For 3-arm RCTs [17, 18], if the null hypothesis that the intervention groups did not differ (z test at 5% significance level) couldn’t be rejected, all groups within the study were pooled and PRP group was defined as intervention while others were defined as control group; Besides, when PRP combined with other treatment methods served as the intervention group and the study was divided into more than 2 groups, the group applied the same standard treatment in PRP group as well as PRP group would be pooled for analysis. The heterogeneity of the studies used the I^2^ statistic, which evaluated the consistency of study results. The cut-off for defining heterogeneity was I^2^ > 50% [19]. If the significant heterogeneity was observed then a random-effects model was used. Otherwise, a fixed-effects model was used. Subgroup analysis were conducted based on intervention method, symptoms duration, BMI, and age. Sensitivity analysis were based on sample size and risk of bias on the overall summary estimates to evaluate whether this restricted analysis affected the magnitude, direction and statistical significance of the overall summary estimate. The strength of evidence was judged by the precision of the CIs, suggesting clinically relevant improvements, and the heterogeneity.

**Fig. 1:**
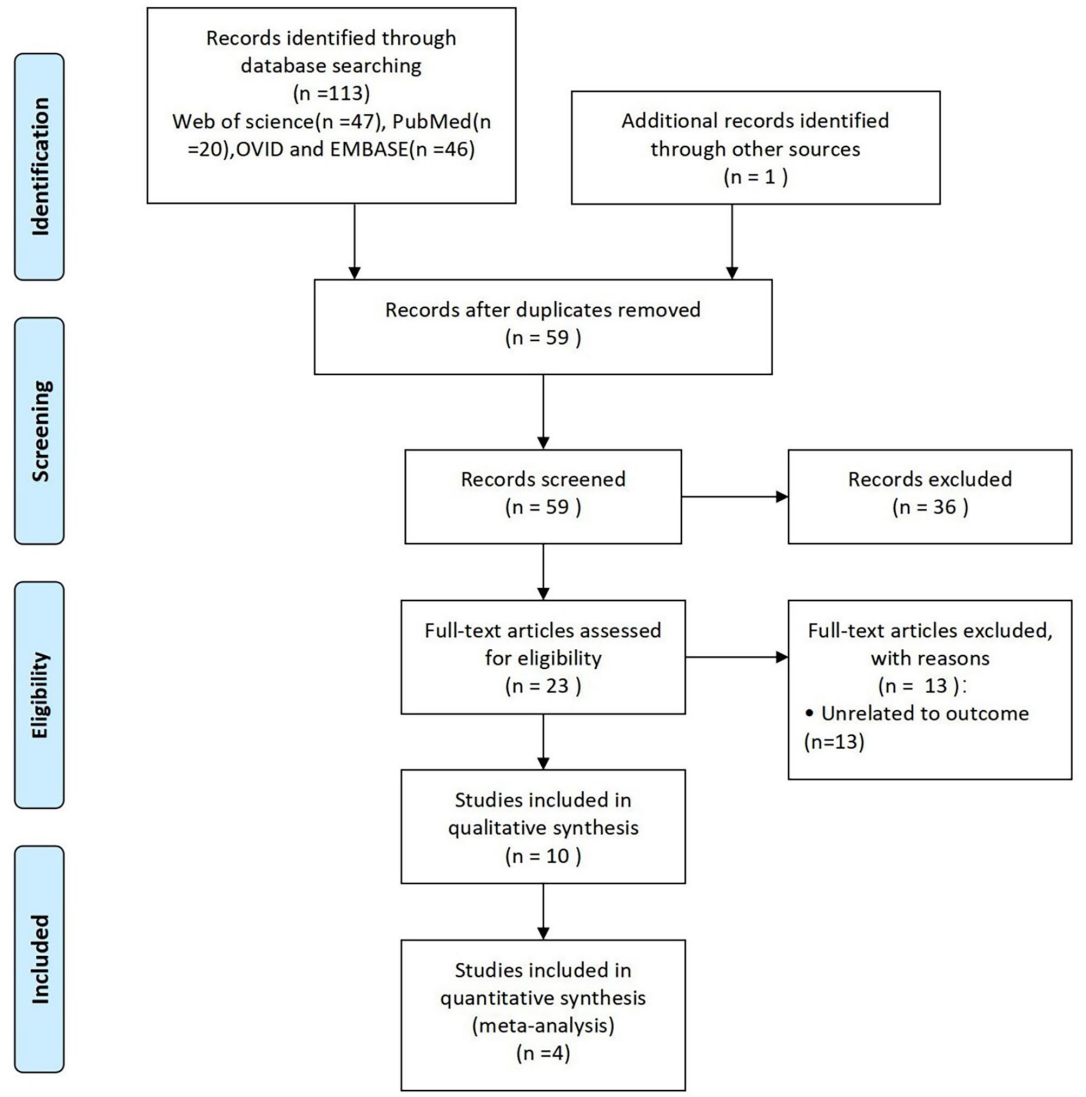
Flow chart for study inclusion and exclusion process

## Results

The database search yielded 113 articles as Fig. 1 showed. After removal of duplicates and irrelevant studies, 10 articles from 7 countries were remained for analysis and 4 articles were into meta-analysis. Three of four RCTs were from Turkey. Six studies [20–25] weren’t into quantitative analysis because they weren’t RCTs, three of which were case series and two were cohort studies, one was controlled studies. Overall, a total of 224 samples were into meta-analysis. Characteristics of each study were showed in Table 1.

**Table 1:** Study details and findings

Author and Year	Country	Study Type, Sample Size	Intervention		Outcomes	Between-group Improvements at Follow-up
			Intervention Group	Control Group		
Guney et al.2016	Turkey	RCT N = 54	Microfracure surgery + PRP	Group 2 (N = 19): Microfracure surgery Group 3 (N = 13): Mosaicplasty	VAS Pain: Improved (*P* < 0.001) AOFAS: Improved (*P* < 0.001) FAAM: Absent baseline data; no intergroup differences at endpoint	No differences between groups at last follow-up; Median 42 months (range: 12–84).
Guney et al.2015	Turkey	Controlled trial N = 35	Microfracure surgery + PRP	Group 2 (N = 16): Microfracure surgery	VAS Pain: Improved (*P* < 0.001) AOFAS: Improved (*P* < 0.001) FAAM: Improved (*P* = 0.001)	Significantly improved at last follow-up as compared to control. Follow-up average of 16.2 months (Range: 12–24)
Görmeli et al.2015	Turkey	RCT N = 40	PRP injection	Group 2 (N = 14): Hyaluronic acid injection Group 3 (N = 13): Saline injection	VAS pain: Improved (*P* < 0.05) AOFAS: Improved (*P* < 0.05) Patient Satisfaction: 61.5% satisfied Adverse Events: None reported	Significantly improved as compared to control, improved patient satisfaction at 1 year. Follow-up average of 15.3 months (range: 11–25).
Paget et al.2021	Netherlands	RCT N = 100	PRP injection	Group 2 (N = 52): Saline injection	AOFAS: Improved (*P* < 0.001) Adverse Events: 1 serious case reported but deemed unrelated to intervention. 13 other adverse events in the PRP group and 8 in the placebo group.	No differences between groups over 26 weeks;
Sampson S et al. 2016	USA	Case Series N = 125 (ankle, N = 6)	Bone marrow concentrate + PRP	No control	VAS pain: Improved Patient Satisfaction: median 9.0/10.0	Follow-up mean 148 days, minimum 56 days.
Fukawa et al. 2017	Japan	Case Series N = 20	PRP injection	No control	VAS pain: Improved (*P* < 0.05) JSSF Ankle/Hindfoot Scale: Improved (*P* < 0.05) SAFE-Q: Improved (*P* < 0.05) Adverse Events: 1 patient had mild pain and swelling resolved within 2 days	Significantly improved VAS and JSSF scores at 4, 12, and 24 weeks. Significantly improved SAFE-Q at 12 weeks.
Li et al. 2021	China	Cohort N = 106	Joint distraction osteogenesis + PRP injection	Group 2 (N = 53): Surgical group	The total effective rate was 98.11% in the combined group and 77.36% in the operation group.	Significant better overall curative effect in PRP group. No significant difference in the incidence of ARs (P > 0.05).
Repetto et al. 2017	Italy	Case Series N = 20	PRP injection	No control	VAS pain: Improved (*P* < 0.05) FADI: Improved (*P* < 0.05) Patient Satisfaction: 80% satisfied Adverse Events: None reported	Significantly improved VAS and FADI at mean 17.7 month follow-up (range: 12–30)
Akpancar et al. 2019	Turkey	Cohort N = 49	PRP injection	Group 2 (N = 27): Prolotherapy injection	AOFAS: Improved (*P* < 0.001) AOS: Improved (*P* < 0.001)	No significant difference between groups at 1 year follow-up.
Mei-Dan et al. 2012	Israel	RCT N = 30	PRP injection	Group 2 (N = 15): Hyaluronic acid injection	AHFS :Improved (*P* < 0.001) VAS pain: Improved *(P* < 0.001)	Significantly improved AHFS at mean 28 weeks follow-up. No between group difference inVAS pain.

RCT: Randomized Controlled Clinical Trial. VAS: Visual Analog Scale. FAAM: Foot and Ankle Ability Measure. AOFAS: American Orthopaedic Foot and Ankle Society scoring system. JSSF: Japanese Society for Surgery of the Foot. SAFE-Q: Self-Administered Foot Evaluation Questionnaire. FADI: Foot and Ankle Disability Index. AOS: Ankle Osteoarthritis Scale. AHFS, Ankle-Hindfoot Scale. Ars: Adverse Reactions.

Among the 10 studies enrolled, 5 were for the talus cartilage injury [17, 18, 22, 25, 26], 4 were for the degenerative osteoarthritis, and 1 was for the post-traumatic osteoarthritis [20]. A total of 4 studies [17, 18, 20, 25] explored the application of PRP as a biological agent to surgery and 3 of which applied PRP after microfracture surgery while 1 of which applied PRP during joint distraction osteogenesis. Another 2 studies [26, 27] explored the effect of PRP applied alone compared to hyaluronic acid (HA) and saline respectively.

**Fig. 2:**
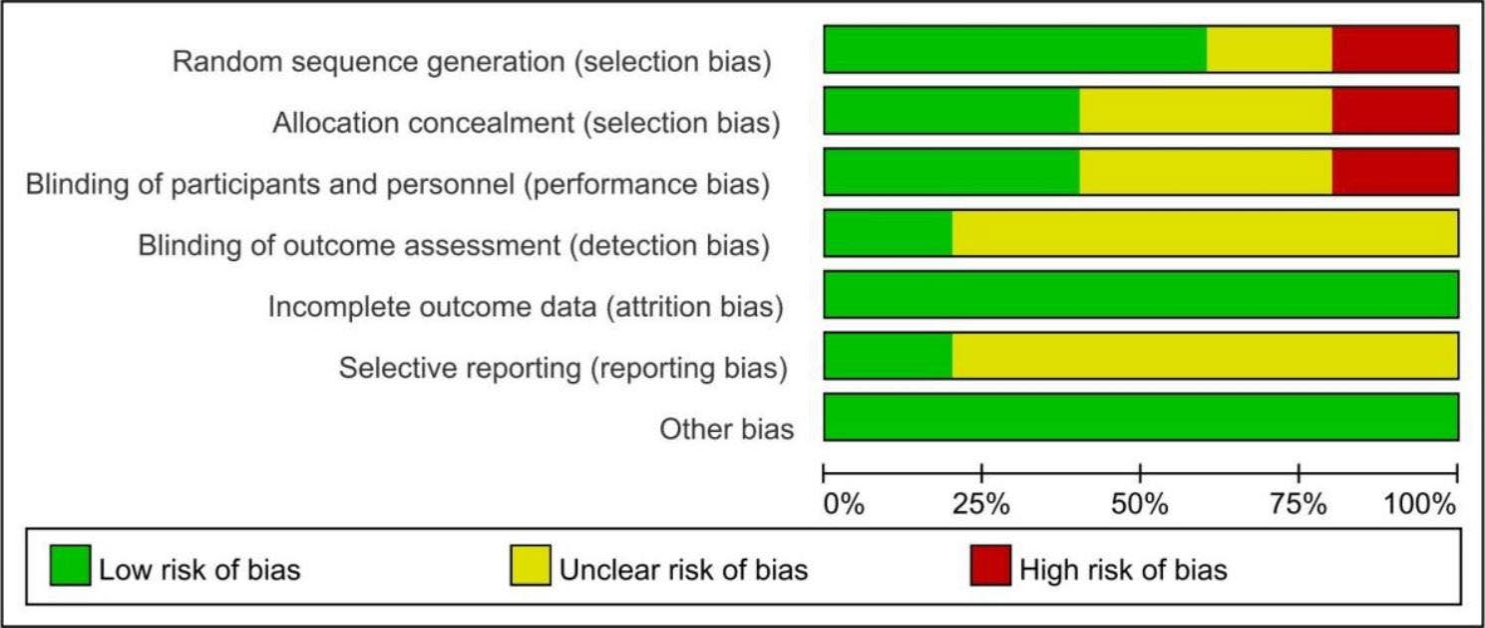
Risk of bias graph for 5 studies

**Fig. 3:**
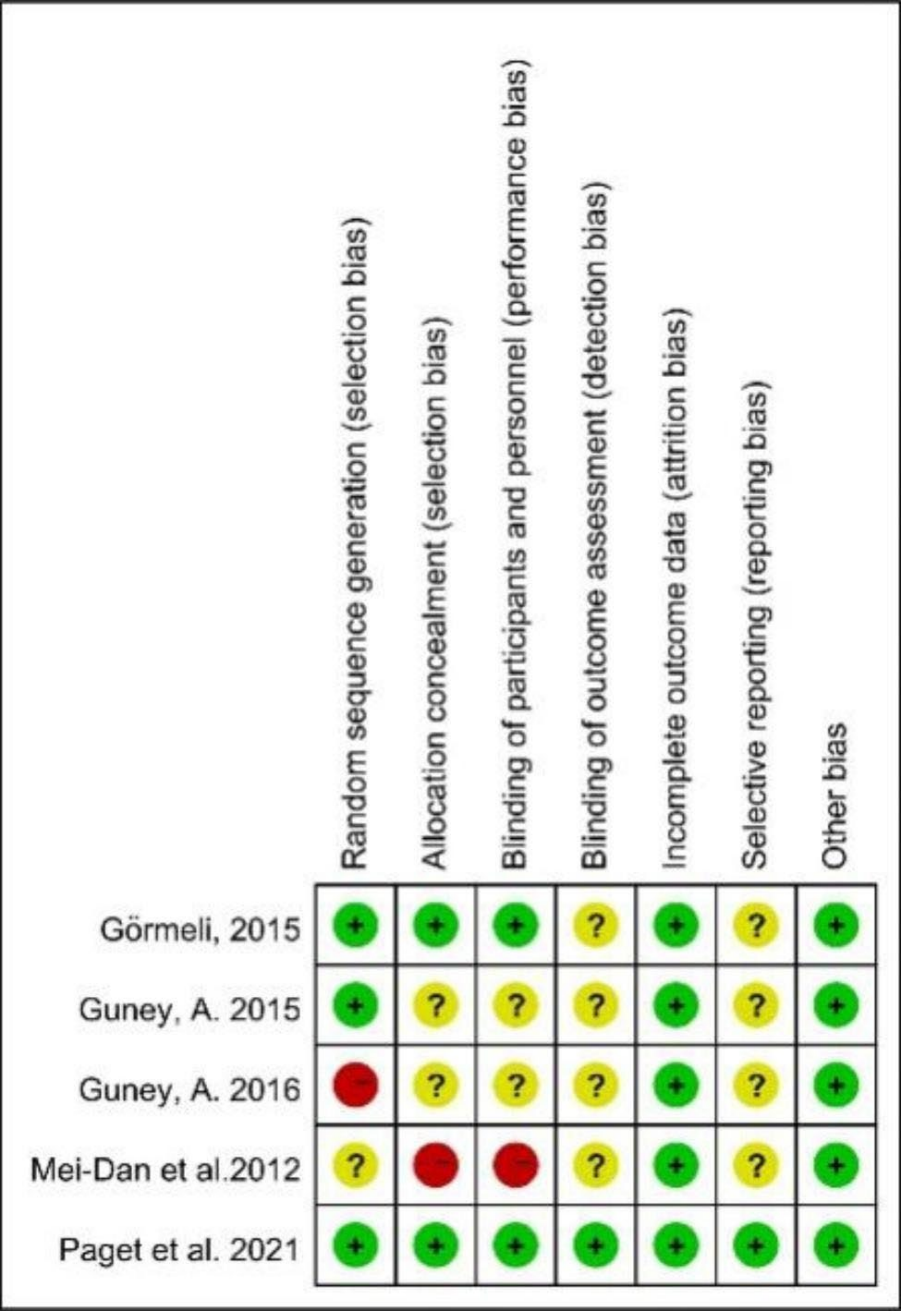
Risk of bias summary for 5 studies

For quality assessment, four RCTs and one controlled study was assessed by the Cochrane Collaboration tool while two cohort studies were assessed by Newcastle-Ottawa Quality Assessment Scale. Other 3 studies were case series. For 3 of all 5 studies, the allocation sequence was adequately generated; in 2 studies, the allocation was adequately concealed and blinding was used (Figs. 2 and 3; Table 2).

**Table 2:** Cohort studies assessed by NOS.

Author and Year	Selection	Comparability	Exposure
Li et al. 2021	★★★★	★	★★★
Akpancar et al. 2019	★★★★	★★	★★★

### Treatment outcome

As shown in Table 1, all studies showed the efficacy of PRP injection for talar cartilage repair, among which 4 studies showed significantly better outcome of PRP group. No missing data related to outcome analysis was reported. Details of PRP preparation and administration of each study were depicted in Table 3.

**Table 3:** PRP preparation and administration

Author and Year	Platelet Concentration vs. Baseline Leukocyte Status	Number of Treatments	Injection Interval	Injection Method		Pre-activation	Centrifugation Procedure
				Dose	Post-procedure management		
Guney et al.2016	6.4 Not Reported	1	-	4mL per time; PRP injected 6–24 h post-microfracture at time of Hemovac drain removal;	Rehabilitation program	Not Reported	Not Reported
Guney et al.2015	6.4 Not Reported	1	-	Dose unknown; PRP injected 6–24 h post-microfracture at time of Hemovac drain removal;	Rehabilitation program	Not Reported	Not Reported
Görmeli et al.2015	5.2 Not Reported	1	-	Dose unknown; PRP injected 24–36 h post-microfracture at time of Hemovac drain removal;	Rehabilitation program	Not Reported	Not Reported
Paget et al.2021	Not Reported Leukocyte-Poor	2	6 weeks	2mL	Education leaflet	Nonactivated	1st centrifugation: 5 min;
Sampson S et al. 2016	4.2 Leukocyte-Poor	1	-	1–2 cc; Bone marrow concentrate was injected intra-articular 8 weeks before PRP;	Rehabilitation program or home exercise program	Nonactivated	1st centrifugation: 2800 rpm for 10 min; 2nd centrifugation: 3400 rpm for 6 min;
Fukawa et al. 2017	5.1 Leukocyte-Poor	3	2 weeks	2mL per time; Ultrasound guided;	Not reported	Calcium chloride	1st centrifugation: 800 g for 5 min; 2nd centrifugation: 1500 g for 8 min;
Li et al. 2021	Not Reported	3	During surgery; 4, 12 weeks after surgery;	5mL per time;	Not reported	Not reported	1st centrifugation: 2000 r / min for 10 min; 2nd centrifugation: 2000 r / min for 10 min;
Repetto et al. 2017	2–3 Leukocyte-Poor	4	1 week	3mL per time;	Not reported	Not reported	1st centrifugation: 3550 rpm for 12 min; 2nd centrifugation: 1100 rpm for 10 min; 3rd centrifugation: 2600 rpm for 20 min;
Akpancar et al. 2019	Not Reported	3	3 weeks	4mL per time; 2 mL for intra-articular and 2 mL for tibial edge and talar dome adjacent to the joint surface;	Not reported	Nonactivated	1st centrifugation: 3200 rpm for 15 min;
Mei-Dan et al. 2012	Not Reported	3	2 weeks	2mL per time;	Avoid unnecessary walking for 24 h. Avoid sports activity or heavy physical work for 2 to 3 days after injection. Avoid nonsteroidal anti-inflammatory medications for 2 weeks after the last injection	Calcium chloride	1st centrifugation: 640 g for 8 min;

For functional outcome measured by AOFAS, the statistical result favored PRP group (MD = 7.84; 95% CI= [-0.13, 15.80], I^2^ = 83%, *P* < 0.01). For pain intensity measured by VAS, the statistical result favored PRP group (MD = 1.86; 95% CI= [0.68, 3.04], I^2^ = 85%, *P* < 0.01). Subgroup analysis showed PRP application method could result in high heterogeneity (Figs. 4 and 5). The application of PRP alone may gain different results from the combined application of PRP and surgery. Guney’s study (2016) and Görmeli (PRP-HA) together remained in sensitivity analysis could significantly reduce heterogeneity for AOFAS (I^2^ = 23%, *P* = 0.26) and VAS (I^2^ = 11%, *P* = 0.29). But none of the factors analyzed by sensitivity were identified as contributors to between-study heterogeneity. It was worth noting that although Guney’s study (2016) follow-up time was the longest, 2 groups of follow-up time differed, which may be one of the sources of heterogeneity.

**Fig. 4:**
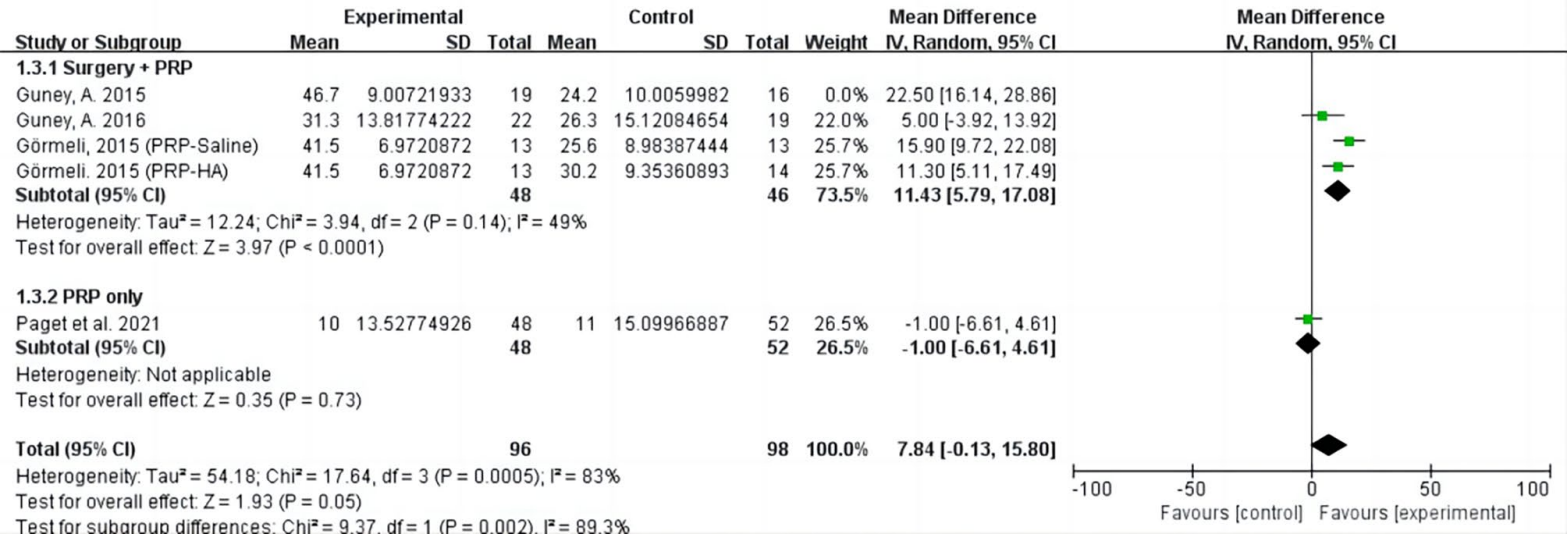
Forest plot of included studies comparing the effect of PRP group and control group on function by AFOAS.

**Fig. 5:**
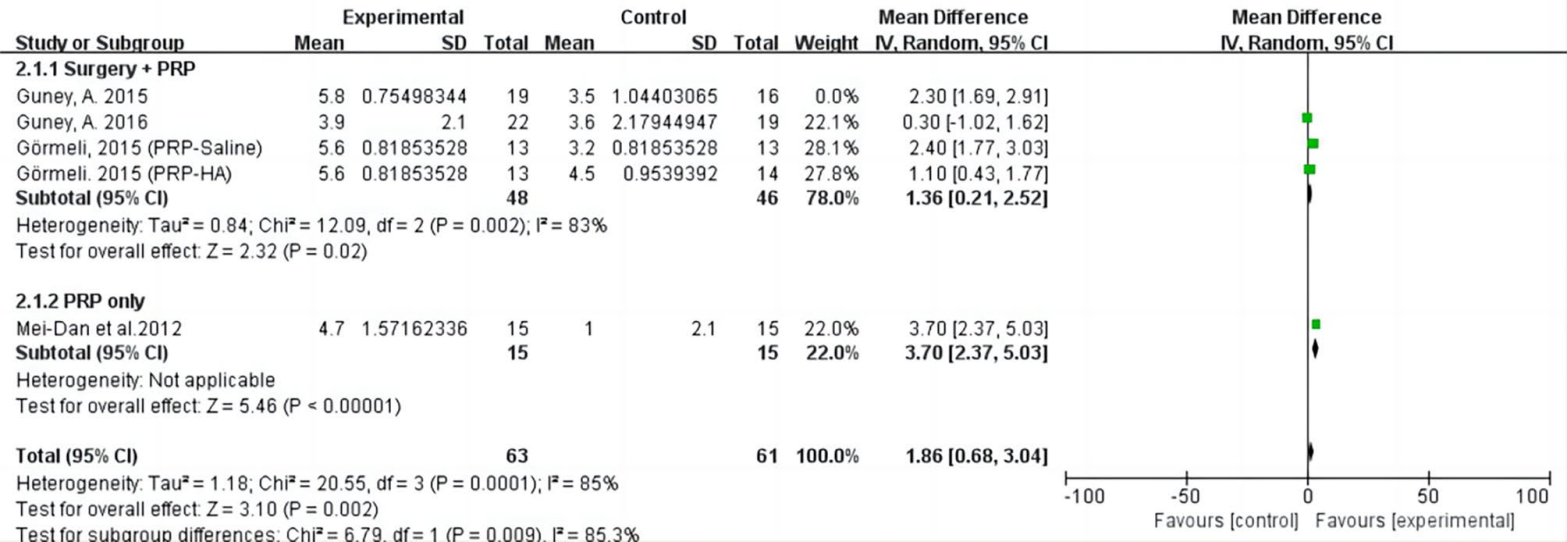
Forest plot of included studies comparing the effect of PRP group and control group on pain by VAS.

Furthermore, the study performed by Sampson [24] et al. also indicated that the intra-articular injection of bone marrow concentrate (BMC) with subsequent application of PRP could lead to more benefits in patients with moderate to severe osteoarthritis. Repetto [21] included grade 3–4 OA patients to find that platelet-rich plasma injection was a valid and safe alternative to postpone the need for surgery with a mean follow-up of 17.7 months. These studies showed a promising effect of PRP to alleviate pain and improve ankle function.

### Adverse events

There were almost no reports of adverse events related to PRP intervention, only Paget [27] et al. reported one case of cerebrovascular disease that was considered to be unrelated to the intervention. It consisted of a transient ischemic attack in the placebo group three weeks after the first injection. At the same time, 13 cases in the PRP group and 8 cases in the control group occurred during the study, which mainly were 2 cases of unilateral knee pain (PRP group) and 19 cases of lower leg muscle soreness (control group, 8 cases). Li [20] et al. reported 2 non-serieous swelling joint while within-group changes of PGE2, TNF-α and IL-6 were all significant (*P* < 0.001).

## Discussion

The systematic review revealed that PRP applied alone or combined with other treatments was safe and effective for the talar cartilage repair in patients with osteoarthritis or talus cartilage injury. There were almost no reports of adverse events related to PRP intervention. As an adjunct to talar-cartilage-related surgery, PRP could improve postoperative function and pain intensity more than saline, HA and non-adjunct. Non-homogeneity of treatments and administration of PRP could result in high heterogeneity. For 4 studies that mentioned post-procedure management, similar phased management was found in 3 meta-studies, meaning that postoperative rehabilitation programs were not impactors of heterogeneity.

The worldwide consensus is that there is still a lack of standardization and classification regarding preparation techniques and clarity in different PRP bioformulations and the related biological properties of the final product are still not conclusive [28]. Therefore, in the follow-up PRP treatment of talus cartilage repair, the study should tend to be standardized. Mentioned apparatus-related factors such as rotational speed are hard to standardize in global applications. However, it may be one of the breakthrough directions to understand the influence of the intrinsic relationship of cytokines contained in different PRP products on the effect of regeneration and repair. It is therefore crucial to investigate the role of the different cytokines and growth factors involved in platelet concentration of PRP, which will facilitate reaching an agreement in application and to guiding PRP preparation and equipment upgrading.

The lack of vascular and lymphatic characteristics contributes to the limited healing ability of articular cartilage [4]. Thus, cartilage metabolism should be taken into account when it comes to regeneration technology. Type II collagen is the main solid component of the extracellular matrix of hyaline cartilage and engages the nourishment of cartilage [4, 7]. A variety of cytokines in PRP could contribute to the expression of excessive type II collagen proteins and proteoglycan [29], promoted chondrocyte differentiation [30], anti-inflammation [28], anti-cartilage catabolism, correction of pathological angiogenesis in osteoarthritis [31–33] and so on. Most studies in this meta-analysis used PRP combined with surgery as treatment, leading to more Type I collagen proliferation which differs from Type II collagen biomechanically [34]. The coverage of the cartilage injury surface may be responsible for the improvement of function and pain intensity. In brief, PRP possibly improves ankle function and pain intensity in mainly two ways: anti-inflammation and promoting cartilage repair. Evans [30] et al. pointed out that PRP was more advantageous in the long-term follow-up of pain symptoms. However, due to the lack of thorough research on specific pathways, it is still controversial whether the effect of PRP in repairing talus cartilage comes from delaying the process of cartilage degeneration or repairing cartilage. More basic research is needed in the future.

### Implications for practice

Firstly, the efficacy of PRP applied alone and in combination with other treatments needs to be studied separately. Secondly, surgery is currently the main combination treatment and there is almost no relevant research to explore the effect of physical therapy combined with PRP treatment on talus cartilage repair which is worth exploring. Thirdly, in the PRP combined with surgical treatment of talus cartilage, how to induce MSCs (Mesenchymal Stem Cell) to differentiate into hyaline cartilage or more type II collagen-containing fibrocartilage is worth exploring. As the same to studies included, other vivo studies have demonstrated that after microfracture, BMC or even autologous chondrocyte implantation, a mechanically inferior type I/II collagen-containing fibrocartilage formed is the most common non-hyaline tissue [7, 35, 36] which may change the ankle force transferring due to different biomechanical properties comparing to type II collagen. In the case of microfracture or BMC, MSCs are the only cell precursor of chondrocytes and their presence within the bone marrow can be as low as 0.001% [37]. Sampson [22] et al. verified that PRP and PDGF may recruit mesenchymal stem cells and enhance the osteogenic potential of MSCs and BMC. The influence pathway and interaction of these growth factors are the key factors and it is possibly the breakthrough direction of PRP combined with various surgical treatments for talus cartilage injury. Additionally, cartilage is tissue with low oxygen tension due to its lack of blood supply. Hypoxia can affect the formation of OA and the degree of cartilage differentiation [38, 39], so whether arthroscopic surgery or intra-articular injection has a certain impact on the level of joint oxygen and thus change the regenerative results is unknown.

### Strengths and limitations

Strengths of this study include a comprehensive search, duplicate assessment of eligibility and data extraction, appraisal of risk of bias, appropriate outcome measurement instruments. To increase the precision of estimates, subgroup analysis and sensitivity analysis were conducted whenever possible. This paper reviews the preparation methods, core parameters and application parameters of PRP promoting talar cartilage repair in different studies, and makes a preliminary summary of the possible mechanism of PRP promoting talar cartilage repair. The quality of the included literature for data synthesis is level I-II with other studies serving as result support and further analysis. Thus, the research outcome is reliable. Limitations of this review are largely the limited available literature, including non-homogeneity of treatments and administration of PRP. Firstly, this review couldn’t distinguish the effects of different PRP dosage, different application frequency, whether anticoagulant or activator was used, whether PRP was prepared at one time, and the temperature conditions for storing PRP on the quality of PRP. Secondly, a small sample size may result in biased results and limited data provided. Thirdly, the degree of injury was different. These studies couldn’t help confirm whether the location of lesions, sizes were comparable and whether they had an impact on the results. Although this article incorporates literature related to talus cartilage repair, studies targeting ankle OA patients did not present a relationship between the course of OA and the history of cartilage damage. Additionally, no worthy factor was identified for the strong heterogeneity of the study. More studies are still needed for further analysis.

## Conclusion

PRP is safe and effective for talar cartilage repair. In addition to the standardization of PRP preparation and application, it is necessary to distinguish the effects of PRP used alone or in combination with other treatments. In PRP studies, surgical treatment of talar cartilage repair remains the mainstream. The regulation of PRP in the surgical application is worth exploring among which the most relative component is MSCs because it is the only exposed chondrocyte precursor in the articular cavity whether it is microfracture or cell transplantation.

## Data Availability

All data generated or analysed during this study are included in this published article.The study was registered in the PROSPERO International prospective register of systematic reviews (CRD42022360183). The protocol was not accessible. Amendments were conducted according to actual condition. Apart from age, subgroup analysis was conducted additionally based on intervention method, symptoms duration, BMI. Sensitivity analysis was also conducted.

## List of abbreviations

PRP: Platelet rich plasma
OLT: Osteochondral lesions of the talus
OA: Osteoarthritis
AOFAS: The American Orthopedic Foot and Ankle Society score
VAS: The Visual Analog Scale
IL-1: Inter-leukin-1
TNFα: Tumor necrosis factorα
MMPs: Matrix metalloproteinases
BMI: Body mass index
BMC: Bone marrow concentrate
MSCs: Mesenchymal Stem Cell
